# Oropouche Virus Disease Among U.S. Travelers — United States, 2024

**DOI:** 10.15585/mmwr.mm7335e1

**Published:** 2024-09-05

**Authors:** Andrea Morrison, Jennifer L. White, Holly R. Hughes, Sarah Anne J. Guagliardo, Jason O. Velez, Kelly A. Fitzpatrick, Emily H. Davis, Danielle Stanek, Edgar Kopp, Peter Dumoulin, Timothy Locksmith, Lea Heberlein, Rebecca Zimler, Joshua Lassen, Carolina Bestard, Edhelene Rico, Alvaro Mejia-Echeverri, Kay-Anna Edwards-Taylor, Douglas Holt, Dionisia Halphen, Kaitlynn Peters, Cheryl Adams, Amanda M. Nichols, Alexander T. Ciota, Alan P. Dupuis, P. Bryon Backenson, Jennifer A. Lehman, Shelby Lyons, Hannah Padda, Roxanne C. Connelly, Van T. Tong, Stacey W. Martin, Amy J. Lambert, Aaron C. Brault, Carina Blackmore, J. Erin Staples, Carolyn V. Gould

**Affiliations:** ^1^Bureau of Epidemiology, Florida Department of Health; ^2^Bureau of Communicable Disease Control, New York State Department of Health; ^3^Division of Vector-Borne Diseases, National Center for Emerging and Zoonotic Diseases, CDC; ^4^Bureau of Public Health Laboratories, Florida Department of Health, Tampa, Florida; ^5^Florida Department of Health in Miami-Dade County, Miami, Florida; ^6^Florida Department of Health in Hillsborough County, Tampa, Florida; ^7^Florida Department of Health in Polk County, Bartow, Florida; ^8^Florida Department of Health in Orange County, Orlando, Florida; ^9^Florida Department of Health in Lee County, Fort Myers, Florida; ^10^Florida Department of Health in Sarasota County, Sarasota, Florida; ^11^Arbovirus Laboratory, Wadsworth Center, New York State Department of Health, Slingerlands, New York; ^12^Epidemic Intelligence Service, CDC; ^13^Division of Birth Defects and Infant Disorders, National Center on Birth Defects and Developmental Disabilities, CDC; ^14^Division of Disease Control and Health Protection, Florida Department of Health.

**Summary**

**What is already known about this topic?**

Oropouche virus is an emerging arthropod-borne virus in the Americas. Recent reports of outbreaks in areas without previous endemic transmission, fatal cases, and vertical transmission associated with adverse pregnancy outcomes have raised concerns about human health risks.

**What is added by this report?**

As of August 16, 2024, a total of 21 Oropouche virus disease cases among U.S. travelers returning from Cuba have been reported. Most patients had self-limited illness. At least three patients experienced recurrent symptoms after resolution of the initial illness.

**What are the implications for public health practice?**

Clinicians and public health jurisdictions should be aware of the occurrence of Oropouche virus disease in U.S. travelers and request testing for suspected cases. Travelers should prevent insect bites when traveling, and pregnant persons should consider deferring travel to areas experiencing outbreaks of Oropouche virus disease.

## Abstract

Beginning in late 2023, Oropouche virus was identified as the cause of large outbreaks in Amazon regions with known endemic transmission and in new areas in South America and the Caribbean. The virus is spread to humans by infected biting midges and some mosquito species. Although infection typically causes a self-limited febrile illness, reports of two deaths in patients with Oropouche virus infection and vertical transmission associated with adverse pregnancy outcomes have raised concerns about the threat of this virus to human health. In addition to approximately 8,000 locally acquired cases in the Americas, travel-associated Oropouche virus disease cases have recently been identified in European travelers returning from Cuba and Brazil. As of August 16, 2024, a total of 21 Oropouche virus disease cases were identified among U.S. travelers returning from Cuba. Most patients initially experienced fever, myalgia, and headache, often with other symptoms including arthralgia, diarrhea, nausea or vomiting, and rash. At least three patients had recurrent symptoms after the initial illness, a common characteristic of Oropouche virus disease. Clinicians and public health jurisdictions should be aware of the occurrence of Oropouche virus disease in U.S. travelers and request testing for suspected cases. Travelers should prevent insect bites when traveling, and pregnant persons should consider deferring travel to areas experiencing outbreaks of Oropouche virus disease. 

## Investigation and Results

### Natural History and Clinical Symptoms

Oropouche virus (Simbu serogroup, genus *Orthobunyavirus*) is endemic to the Amazon region and was previously identified as a cause of human disease in several countries in South and Central America and the Caribbean (*1*). The virus circulates in a sylvatic cycle, possibly involving certain vertebrate hosts (e.g., sloths, nonhuman primates, and birds) and mosquitoes, and an urban cycle in which humans serve as amplifying hosts with known vectors being biting midges (*Culicoides paraensis*) and possibly mosquitoes (e.g., *Culex quinquefasciatus*) (*1*).

The clinical signs and symptoms of Oropouche virus disease are similar to those of other arboviral diseases such as dengue, Zika, and chikungunya. After an incubation period of 3–10 days, patients typically experience abrupt onset of fever, chills, headache, myalgia, and arthralgia. Other symptoms might include retroorbital pain, photophobia, vomiting, diarrhea, fatigue, maculopapular rash, conjunctival injection, and abdominal pain. Initial symptoms usually last only a few days, but up to 70% of patients are reported to have recurrent symptoms within days to weeks after resolution of their initial illness (*2*). Although illness is typically mild, hemorrhagic manifestations (e.g., epistaxis, gingival bleeding, melena, menorrhagia, and petechiae) or neuroinvasive disease (e.g., meningitis and meningoencephalitis) can rarely occur (*1**,**3**,**4*). No vaccines to prevent or medicines to treat Oropouche virus disease exist; treatment is supportive.

### Recent Outbreaks in South America and Cuba

During December 2023–June 2024, large Oropouche virus disease outbreaks were recognized in areas with known endemic disease, and the virus emerged in new areas in South America and Cuba where it had not been historically reported (*3*). As of August 2024, over 8,000 laboratory-confirmed cases have been reported in Bolivia, Brazil, Colombia, Cuba, and Peru (*3*). These large outbreaks have resulted in travel-associated cases, with 19 Oropouche virus disease cases in European travelers returning from Cuba (n = 18) and Brazil (one) during June–July 2024 (*5*). Recently, cases of severe disease leading to two deaths and vertical transmission associated with fetal death and possible congenital malformations in Brazil have raised concerns about the threat of Oropouche virus to human health (*3*).

### Identification of U.S. Cases

CDC and New York State Department of Health (NYSDOH) Wadsworth Center conducted Oropouche virus testing for travelers who had returned from areas with known Oropouche virus circulation and had an illness that was clinically compatible with Oropouche virus disease. Clinical diagnostic testing at CDC’s Arboviral Diseases Branch and NYSDOH Wadsworth Center Arbovirus Laboratory is performed using a 90% plaque reduction neutralization test (PRNT_90_) to detect virus-specific neutralizing antibodies in serum or cerebrospinal fluid, with titers ≥10 considered positive. CDC also conducted surveillance testing on specimens collected ≤7 days after symptom onset using an Oropouche virus real-time reverse transcription–polymerase chain reaction (RT-PCR) assay (*6*). This activity was reviewed by CDC, deemed not research, and was conducted consistent with applicable federal law and CDC policy.*

The Florida Department of Health (FLDOH) identified suspected cases primarily by reviewing patients who received negative test results for dengue from state and commercial laboratories and who had a clinically compatible illness and exposure to areas with potential Oropouche virus circulation. Details of epidemiologic investigations, including risk factors, clinical features, and outcomes, are captured from patient interview, clinician interview, or review of medical records using a standardized case investigation form.

### Characteristics of U.S. Cases

Evidence of Oropouche virus infection was identified in 21 U.S. residents returning from travel to Cuba, including 20 in Florida and one in New York. Most patients were initially evaluated during their acute illness, but at least three patients were evaluated when their symptoms reoccurred after initial symptom resolution. The median patient age was 48 years (range = 15–94 years) and 48% were female (Table 1). Pregnancy status was not included in this report for reasons of confidentiality. Reported symptoms commenced during May–July and most commonly included fever (95%), myalgia (86%), headache (76%), fatigue or malaise (62%), and arthralgia (57%). Other reported signs and symptoms included diarrhea (48%), abdominal pain (29%), nausea or vomiting (29%), rash (29%), retroorbital pain (24%), back pain (19%), and mucosal bleeding (5%) (Table 2). The combination of fever and myalgia with or without other symptoms was reported in 17 (81%) patients; the combination of fever and headache was reported in 15 (71%). All three symptoms occurred in 13 (62%) patients. Overall, three were hospitalized, and no deaths were reported.

**TABLE 1:** Characteristics of U.S. travelers with Oropouche virus disease (N = 21) — United States, 2024

Characteristic	No. (%)
**Age group, yrs**	
0–19	2 (10)
20–39	5 (24)
40–59	10 (48)
≥60	4 (19)
**Sex**	
Female	10 (48)
**State of residence**	
Florida	20 (95)
New York	1 (5)
**Location of travel**	
Cuba	21 (100)
**Symptom onset, month**	
May	1 (5)
June	6 (29)
July	14 (67)

**TABLE 2:** Signs and symptoms* reported by U.S. travelers with Oropouche virus disease (N = 21) — United States, 2024

Patient	Sign or symptom											
	Fever	Myalgia	Headache	Fatigue/ Malaise	Arthralgia	Diarrhea	Retroorbital pain	Abdominal pain	Nausea/ Vomiting	Back pain	Rash	Mucosal bleeding
A	X	X	X	X	X	—	X	—	X	X	—	—
B	X	X	X	X	X	—	X	—	—	—	X	—
C	X	X	X	X	X	—	—	—	—	X	X	—
D	X	X	X	X	X	X	—	X	—	—	—	—
E	X	X	X	X	—	X	—	X	X	—	—	—
F	X	X	X	X	—	X	—	X	—	X	—	—
G	X	X	X	X	—	—	—	—	—	—	—	—
H	X	X	X	—	X	—	—	—	X	—	—	—
I	X	X	X	—	X	—	—	X	—	—	—	X
J	X	X	X	—	—	—	—	—	X	—	—	—
K	X	X	X	—	—	X	—	—	—	—	X	—
L	X	X	X	—	—	—	—	—	—	—	—	—
M	X	X	X	—	—	—	—	—	—	—	—	—
N	X	X	—	X	X	—	—	—	—	X	X	—
O	X	X	—	X	X	X	—	—	—	—	—	—
P	X	X	—	—	X	X	X	X	—	—	—	—
Q	X	X	—	—	X	X	—	—	—	—	X	—
R	X	—	X	X	—	X	X	X	X	—	—	—
S	X	—	X	X	X	X	—	—	—	—	—	—
T	X	—	—	X	—	X	—	—	—	—	X	—
U	—	X	X	X	X	—	X	—	X	—	—	—
**Total no. (%) reporting sign or symptom**	**20 (95)**	**18 (86)**	**16 (76)**	**13 (62)**	**12 (57)**	**10 (48)**	**5 (24)**	**6 (29)**	**6 (29)**	**4 (19)**	**6 (29)**	**1 (5)**

* Within cells, X = sign or symptom reported; dash = no sign or symptom reported.

Laboratory evidence of Oropouche virus infection was identified by real-time RT-PCR in 13 patients, by PRNT_90_ in seven, and by both assays in one patient. Most real time RT-PCR–positive specimens were collected on days 1–4 (median = 2.5 days; range = 1–7 days) after symptom onset. PRNT_90_–positive specimens were collected a median of 17 days (range = 9–32 days) after symptom onset.

## Public Health Response

As a result of the emergence and spread of Oropouche virus in the Americas, CDC is working with state public health jurisdictions and international partners to enable rapid detection and surveillance of Oropouche virus transmission and disease to guide public health prevention measures. CDC is currently developing a plan for rapid detection and response to Oropouche virus disease cases in the United States, assisting health departments with clinical diagnostic and surveillance testing for suspected cases, working to validate a molecular assay to detect acute infections, and updating CDC’s Travelers’ Health notices^†^ and website^§^ on Oropouche as new information becomes available. In addition, CDC is providing clinical consultation and guidance to pregnant persons and their care providers and are tracking the impact of emerging health threats, like Oropouche virus, on pregnant persons and their infants.^¶^ Although Oropouche virus disease is not nationally notifiable, CDC encourages jurisdictions to report cases voluntarily to ArboNET, the national arboviral disease surveillance system, using interim case definitions.** For questions about testing or reporting, health departments can contact eocevent495@cdc.gov.

## Discussion

The 21 U.S. travel-associated Oropouche virus disease cases were all identified among U.S. residents who had traveled to Cuba. The clinical features of the travelers’ illnesses are similar to those reported in the literature (*1**,**4**,**7*). Most patients had a self-limited febrile illness, commonly associated with myalgia and headache with or without additional signs or symptoms, including gastrointestinal symptoms (reported by approximately two thirds of patients). At least three patients initially sought care after experiencing relapse of symptoms following resolution of the initial illness. This reported reoccurrence of symptoms is unique to Oropouche virus disease and is not typically reported in cases of similar arboviral diseases, such as dengue or Zika virus disease (*2*). The reoccurrence of symptoms is likely underestimated because of limitations in obtaining a complete clinical history or follow-up after the initial illness.

Among most patients, Oropouche virus disease is mild; however, two deaths in previously healthy young persons with Oropouche virus infection were recently reported in Brazil (*3*). In July, the Pan American Health Organization (PAHO) issued an epidemiologic alert concerning possible vertical transmission of Oropouche virus disease associated with adverse pregnancy outcomes, including fetal deaths and congenital malformations (*3*).

Clinicians should report suspected Oropouche virus disease cases to state, tribal, local, or territorial health departments to facilitate testing and implementation of community prevention measures and messaging.^††^ Information for health care providers regarding clinical features, diagnosis, and clinical management are available on CDC’s website.^§§^ Supportive care is recommended for clinical management of patients. Patients should be advised to avoid nonsteroidal anti-inflammatory drugs to reduce the risk for bleeding. Oropouche and dengue viruses can cocirculate and cause similar symptoms; patients with clinically suspected dengue should be managed according to dengue clinical management recommendations^¶¶^ until dengue is ruled out. Interim considerations for clinical management of pregnant persons with Oropouche virus disease and infants born to these pregnant persons are available.***

Oropouche virus disease should be considered in a patient who has been in an area with documented or suspected Oropouche virus circulation (*3*) within 2 weeks of initial symptom onset and who experiences an abrupt onset of fever, headache, and one or more of the following: myalgia, arthralgia, photophobia, retroorbital or eye pain, or signs and symptoms of neuroinvasive disease (e.g., stiff neck, altered mental status, seizures, limb weakness, or cerebrospinal fluid pleocytosis). Because patients with Oropouche virus disease can experience reoccurrence of symptoms after resolution of the initial illness, patients might seek care >2 weeks after travel. In suspected Oropouche virus disease cases, testing should be conducted for other diseases with similar symptoms, including dengue, particularly given the recent large dengue outbreak in the Americas with approximately 11 million cases reported since late 2023 (*8*). Because of the concern for vertical transmission of Oropouche virus from a pregnant patient to the fetus, paired specimens should be collected from pregnant patients to confirm a recent infection.

### Implications for Public Health Practice

Guidance on clinical case identification and management might be modified as the epidemiologic situation evolves, particularly if local transmission in the United States is identified and as more is learned about disease and transmission risk. Based on presently available data, the risk for sustained local transmission in the continental United States is likely low, whereas the risk for sustained transmission in Puerto Rico and U.S. Virgin Islands is unknown. CDC is working with partners to understand more about what is driving the current outbreaks and how that might affect risk of transmission. Vector competence studies are underway to understand the potential role of several U.S. *Culicoides* spp. of biting midges and mosquito species (*Cx. quinquefasciatus* and *Aedes aegypti)* in Oropouche virus transmission.

Providers should advise persons of the risk for Oropouche virus disease and counsel them to use personal protective measures^†††^ against mosquito and biting midge bites if traveling to areas with virus circulation. Travelers should use personal protective measures for 3 weeks after return from an area with Oropouche virus circulation, or during the first week of illness in symptomatic patients to prevent further spread, especially in areas where mosquitoes or biting midges are active. Because of the risk for possible vertical transmission providers should inform persons who are pregnant and considering travel to areas with reported Oropouche virus transmission of the possible risks to the fetus. Pregnant travelers should prevent insect bites during travel^§§§^ and consider deferring travel to areas experiencing outbreaks of Oropouche virus disease.^¶¶¶^ CDC is working with PAHO and other partners to learn more about the potential risks associated with infection with Oropouche virus during pregnancy and to increase testing capacity in the region.

## Footnotes

* 45 C.F.R. part 46.102(l)(2), 21 C.F.R. part 56; 42 U.S.C. Sect. 241(d); 5 U.S.C. Sect. 552a; 44 U.S.C. Sect. 3501 et seq.

† https://wwwnc.cdc.gov/travel/notices

§ https://www.cdc.gov/oropouche/about/index.html

¶ https://www.cdc.gov/set-net/about/index.html

**
https://www.cdc.gov/oropouche/php/reporting/index.html

††
https://emergency.cdc.gov/han/2024/han00515.asp

§§
https://www.cdc.gov/oropouche/hcp/clinical-overview/index.html

¶¶ https://www.cdc.gov/dengue/hcp/clinical-care/index.html

*** https://www.cdc.gov/oropouche/hcp/clinical-care-pregnancy/index.html; https://www.cdc.gov/oropouche/hcp/clinical-care/infants.html

††† https://www.cdc.gov/oropouche/prevention/index.html

§§§
https://www.cdc.gov/mosquitoes/prevention/preventing-mosquito-bites-while-traveling.html

¶¶¶ https://wwwnc.cdc.gov/travel/notices/level2/oropouche-cuba
